# Low CDKN1B Expression Associated with Reduced CD8+ T Lymphocytes Predicts Poor Outcome in Breast Cancer in a Machine Learning Analysis

**DOI:** 10.3390/jpm14010030

**Published:** 2023-12-25

**Authors:** Hyung-Suk Kim, Yung-Kyun Noh, Kyueng-Whan Min, Dong-Hoon Kim

**Affiliations:** 1 Division of Breast Surgery, Department of Surgery, Hanyang University Guri Hospital, Hanyang University College of Medicine, Guri 15588, Republic of Korea; hyung6960@hanyang.ac.kr; 2 Department of Computer Science, Hanyang University, 222 Wangsimni-ro, Seoul 04763, Republic of Korea; nohyung@hanyang.ac.kr; 3 School of Computational Sciences, Korea Institute for Advanced Study, Seoul 02455, Republic of Korea; 4 Department of Pathology, Uijeongbu Eulji Medical Center, School of Medicine, Eulji University, Uijeongbu 11759, Republic of Korea; 5 Department of Pathology, Kangbuk Samsung Hospital, School of Medicine, Sungkyunkwan University, 29 Saemunanro, Seoul 03181, Republic of Korea

**Keywords:** breast cancer, prognosis, tumor infiltrating lymphocyte, CDKN1B

## Abstract

The cyclin-dependent kinase inhibitor 1B (CDKN1B) gene, which encodes the p27Kip1 protein, is important in regulating the cell cycle process and cell proliferation. Its role in breast cancer prognosis is controversial. We evaluated the significance and predictive role of CDKN1B expression in breast cancer prognosis. We investigated the clinicopathologic factors, survival rates, immune cells, gene sets, and prognostic models according to CDKN1B expression in 3794 breast cancer patients. We performed gene set enrichment analysis (GSEA), in silico cytometry, pathway network analyses, gradient boosting machine (GBM) learning, and in vitro drug screening. High CDKN1B expression levels in breast cancer correlated with high lymphocyte infiltration signature scores and increased CD8+ T cells, both of which were associated with improved prognosis in breast cancer. which were associated with a better prognosis. CDKN1B expression was associated with gene sets for the upregulation of T-cell receptor signaling pathways and downregulation of CD8+ T cells. Pathway network analysis revealed a direct link between CDKN1B and the pathway involved in the positive regulation of the protein catabolic process pathway. In addition, an indirect link was identified between CDKN1B and the T-cell receptor signaling pathway. In in vitro drug screening, BMS-345541 demonstrated efficacy as a therapeutic targeting of CDKN1B, effectively impeding the growth of breast cancer cells characterized by low CDKN1B expression. The inclusion of CDKN1B expression in GBM models increased the accuracy of survival predictions. CDKN1B expression plays a significant role in breast cancer progression, implying that targeting CDKN1B might be a promising strategy for treating breast cancer.

## 1. Introduction

Breast cancer is one of the most commonly diagnosed cancers in women, and recent advances in early detection and a variety of systemic and targeted therapies have improved survival rates for breast cancer patients. Breast cancer is still one of the primary causes of death from cancer, and the rate of decline in mortality has decelerated in recent years [1]. Breast cancer has a heterogeneous nature and exhibits genetic and phenotypic diversity, which may appear clinically or pathologically similar but demonstrate different behaviors due to underlying biological differences [2,3]. Therefore, to improve overall survival, it is essential to understand the molecular mechanisms associated with breast cancer progression.

The cyclin-dependent kinase inhibitor 1B (CDKN1B) is critical for the regulation of the cell cycle and cell proliferation. It encodes p27, a cyclin-dependent kinase inhibitor (CDKi) in the Kip family of cyclin-dependent kinase inhibitors. p27 functions as a tumor suppressor because it inhibits the activity of cyclin-dependent kinases (CDKs) and prevents cell division, thereby inhibiting the transition from the G1 to the S phase [4,5]. Downregulation or inactivation of CDKN1B resulting from genetic mutations, modifications, or dysregulation of the signaling pathway can impair normal function. Consequently, it may promote human tumorigenesis or oncogenic development in numerous human malignancies, including breast cancer, by causing uncontrolled cell growth [5,6,7].

Advances in next-generation sequencing (NGS) and genome-wide association analysis have revealed many potential factors that promote tumor progression in various cancers. Among these, mutations in CDKN1B have been shown to occur at a frequency of 2.8% in breast cancer patients [8]. Mutations in CDKN1B have been discovered and confirmed to be most significantly altered in luminal breast cancer, a subtype that accounts for more than 60% of all breast cancers, and high CDKN1B expression has been demonstrated to predict sensitivity to endocrine therapy and chemotherapy in luminal breast cancer patients [9,10]. The molecular processes driving carcinogenesis and the clinicopathological variances associated with CDKN1B expression in breast cancer remain incompletely understood. Understanding the molecular mechanisms underlying CDKN1B dysregulation and its impact on breast cancer progression is important to improve treatment strategies for breast cancer by developing potential therapeutic targets and identifying its role as a prognostic marker. In recent years, bioinformatics and high-throughput experimental analyses have utilized multi-omic data obtained from marker genes, quantification of tumor-infiltrating immune cells, and molecular networks to identify reliable biomarkers essential for successful treatment approaches.

**Figure 1:**
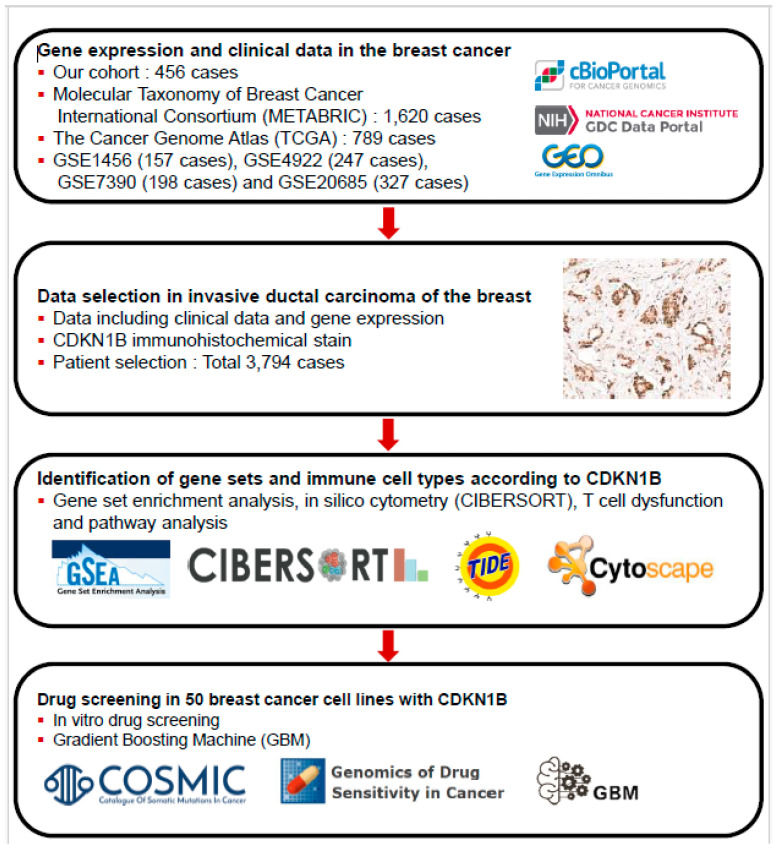
Schematic diagram depicting the plan of the study.

In this study, our objective was to examine the correlation between CDKN1B expression levels and clinicopathological factors as well as survival rates in breast cancer patients. We analyzed the effect of CDKN1B expression on breast cancer survival using the gradient boosting machine (GBM) algorithm. Additionally, we conducted gene set enrichment analysis (GSEA) and pathway network analysis to investigate the gene sets associated with CDKN1B expression. This investigation revealed the underlying mechanism of CDKN1B expression in breast cancer by understanding the anticancer immune responses associated with these gene sets. To identify potential effective drug targets for breast cancer cell lines with low CDKN1B expression, the Genomics of Drug Sensitivity in Cancer (GDSC) database was used as an in vitro drug screening platform (Figure 1).

## 2. Materials and Methods

### 2.1. Patient Selection

The study included 456 breast cancer patients with invasive ductal carcinoma whose primary tumors were operated on at both Hanyang University Guri Hospital and Samsung Kangbuk Medical Center. Patients with unavailable clinicopathological parameters or missing tissue blocks were excluded. We enrolled 1620 breast cancer patients with a pathologic diagnosis of invasive ductal carcinoma from the Molecular Taxonomy of Breast Cancer International Consortium (METABRIC), as well as 789 such patients from The Cancer Genome Atlas (TCGA) [11,12,13]. Gene expression profiling data sets such as GSE1456, GSE4922, GSE7390, and GSE20685 were downloaded from the GEO (gene express omnibus) database (http://www.ncbi.nlm.nih.gov/geo/) (accessed on 1 June 2021) [14,15,16,17]. Survival analyses were performed to determine clinical significance in four independent cohorts, including GSE1456 (N = 157), GSE4922 (N = 247), GSE7390 (N = 198), and GSE20685 (N = 327). Genomic data from 179 normal breast tissue samples were obtained from a resource database, the Genotype-Tissue Expression (GTEx) project [18].

Survival data included disease-free survival (DFS), defined as the time (in months) from the date of diagnosis to recurrence including distant metastasis, and disease-specific survival (DSS), defined as time (in months) from the date of primary surgical treatment to the time of death by breast cancer.

### 2.2. Tissue Microarray Construction and Immunohistochemistry

**Figure 2:**
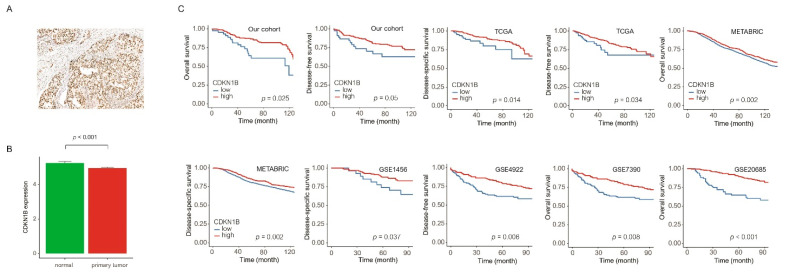
(**A**) Immunohistochemical staining showing CDKN1B expression in breast cancer (original magnification 200×). (**B**) Bar plots of CDKN1B expression, normal tissue versus breast cancer (error bars: standard errors of the mean) (*p* < 0.001). (**C**) Low CDKN1B expression was associated with shorter survival time compared with high CDKN1B expression (all *p* < 0.05).

Tissue microarray (TMA) was prepared using a tissue array device (AccuMax Array; ISU ABXIS Co. Ltd., Seoul, Republic of Korea). We used duplicate 3 mm diameter tissue cores (tumor component in a tissue core > 70%) from each donor block. Sections of four micrometers in size were extracted from the TMA blocks following standard techniques. Immunostaining for p27 protein (encoded by the CDKN1B gene) (clone 1B4, 1:50, Novocastra, Newcastle, UK) was performed using the Dako Autostainer Universal Staining System (DakoCytomation, Carpinteria, CA, USA) and ChemMate™ Dako EnVision™ Detection Kit. By assessing the intensity and proportion of nucleus-stained tumor cells, the immunoreactive score (IRS) for p27 expression was derived [19] (Figure 2A). The level of p27 expression was evaluated via a receiver operating characteristic (ROC) curve and categorized as low (IRS < 1) or high (IRS ≥ 1). Hormone receptor (HR) positive status was defined as positive immunohistochemistry (IHC) for estrogen and/or progesterone receptors (ER/PR). Human epidermal growth factor receptor 2 (HER2)-positive status was defined as an IHC score of 3+ or 2+ accompanied by confirmed HER2 gene amplification by fluorescent in situ hybridization (FISH) or silver in situ hybridization (SISH) [20]. The IHC results have been used for the classification of tumors into different molecular subtypes of breast cancer in our cohort: HR-positive/HER2-negative, HR-positive/HER2-positive, HR-negative/HER2-positive, and HR-negative/HER2-negative [21].

### 2.3. Immunohistochemistry for CD8+ T Cells

Formalin-fixed paraffin-embedded blocks from 172 of the 456 patients in our cohort were sectioned and stained for immunohistochemistry from one of our cohorts. Anti-CD8 (clone 4B11; Leica Biosystems, Newcastle, UK) and anti-CD4 (clone 4B12; Leica Biosystems) antibodies were detected using the Bond Polymer Refine Detection System (Leica Biosystems) according to the manufacturer’s instructions. CD8+ T cell and CD4+ T cell counts were determined avoiding areas of necrosis. In cases of heterogeneity, CD8+ T cell and CD4+ T cell counts were estimated at the tumor front within the area of deepest invasion. A minimum of three random fields were examined to assess both the tumor center and the intraepithelial compartment. In cases of heterogeneity, the count that best represented the entire section was assigned, as previously described [22].

### 2.4. GSEA, In Silico Cytometry, and Pathway Network Analyses

Using gene set enrichment analysis (GSEA, version 4.2.2), we performed a comprehensive analysis of relevant gene sets [23]. To identify gene sets associated with low CDKN1B expression, curated gene sets (C2, 6495 sets), oncogenic gene sets (C6, 189 sets), and immunological gene sets (C7, 5219 sets) were applied. For this study, we performed 1000 permutations to determine *p* values, using a phenotype-based permutation type. The following cutoffs were used: *p* < 0.05 and false discovery rate (FDR) < 0.25. To explore leukocyte subsets, we used in silico cytometry (CIBERSORT) [24]. Pathway network analyses were plotted using Cytoscape software (version 3.9.1) and ClueGO (version 2.5.8), an application for Gene Ontology (GO) analyses. The networks were categorized by considering functionally enriched Gene Ontology (GO) terms and pathways [25,26]. We used the Tumor Immune Dysfunction and Exclusion (TIDE) tool to identify two biomarkers, interferon-γ and TIDE; cancer-associated fibroblasts; and immunotherapy response [27].

### 2.5. Genomics of Drug Sensitivity in Cancer Database and GBMs

We investigated the relationship between sensitivity to anticancer drugs using data from the Genomics of Drug Sensitivity in Cancer (GDSC) dataset [28] and the Cell Lines Project of the Catalog Of Somatic Mutations In Cancer (COSMIC) database [29]. A total of 50 breast cancer cell lines were stratified into high and low groups according to the median CDKN1B expression level. In breast cancer cell lines exhibiting low or high CDKN1B expression, drug response was determined as the natural log of the half-maximal inhibitory concentration (LN IC50). Drug efficacy was determined by a decrease in the calculated LN IC50 in cell lines with low CDKN1B expression and an increase in those with high CDKN1B expression, indicating a positive correlation between drug effect and CDKN1B expression levels [30,31].

To construct prognostic models for survival prediction, we integrated CDKN1B with clinical risk factors including T and N stage, histological grade, perineural invasion, lymphatic invasion, ER, HER2, and CDKN1B. Machine learning algorithms were then applied to a dataset of 456 cases, with 70% randomly assigned as the training set and 30% as the validation set. A machine learning algorithm was used to automatically select and combine several predictors from gradient boosting machines (GBMs) using multivariate Bernoulli models. The performance of the GBM method was evaluated by a receiver operating characteristic (ROC) curve.

### 2.6. Statistical Analysis

The study utilized the χ^2^ test to analyze the correlations between clinicopathological parameters and CDKN1B. Differences among continuous variables were examined using the student’s *t*-test and/or Spearman’s correlation analysis. To compare survival curves, the study employed the Kaplan–Meier method and the log-rank test. Moreover, independent prognostic markers for survival were identified using multivariate Cox regression analyses. A statistical significance level was set at a two-tailed *p*-value of less than 0.05. The analysis of all data was conducted using the R (version 4.0.2, R Foundation for Statistical Computing, Vienna, Austria).

## 3. Results

### 3.1. Clinicopathological Parameters and Survival Analysis of CDKN1B

In our study, the cohort of 456 breast cancer patients was stratified into two categories using the optimal cut-off for CDKN1B expression. Of these patients, 85 (18.6%) were classified as having low CDKN1B expression, while 371 (81.4%) were classified as having high CDKN1B expression. A detailed comparison of the clinicopathological characteristics between the two groups is shown in Table 1. Low CDKN1B expression was significantly associated with advanced T stage (*p* = 0.034), advanced N stage (*p* = 0.021), vascular invasion (*p* = 0.001), and perineural invasion (*p* = 0.008). On the other hand, high CDKN1B expression was significantly associated with HER2 overexpression (*p* = 0.016), higher Ki67 (*p* = 0.001), and higher p53 expression (*p* < 0.001). Within the GTEx and TCGA datasets, a significant decrease in CDKN1B expression was observed in primary tumors compared to normal tissue (*p* < 0.001) (Figure 2B). Among our cohort, individuals with low CDKN1B expression had notably worse disease-free survival (DFS) and overall survival (OS) outcomes than those with high CDKN1B expression (*p* = 0.05 and *p* = 0.025, respectively). We conducted further survival analysis on six large datasets (TCGA, METABRIC, GSE1456, GSE4922, GSE7390, and GSE20685) to validate the level of CDKN1B expression and clinical outcomes. Low CDKN1B expression revealed a significantly shorter survival time than high CDKN1B expression (all *p* < 0.05) (Figure 2C). In our cohort, as well as in the TCGA and METABRIC datasets, the statistical significance persisted in the multivariate analysis (Table 2).

In our cohort, the patients with low CDKN1B expression significantly correlated with poor DFS (univariate, *p* = 0.014; multivariate, *p* = 0.043) and OS (univariate, *p* = 0.003; multivariate, *p* = 0.033) in the HR(+) HER2(−) subtype. In the HR(+) HER2(+), patients with low CDKN1B expression were associated with poor DFS (*p* = 0.012) and OS (*p* = 0.032) only in univariate analysis. No statistically significant correlation was found between CDKN1B expression and survival outcomes for the remaining subtypes (Supplementary Table S1).

### 3.2. Gene Sets, Immune Response, and Pathway Network Analysis According to CDKN1B Expression

**Figure 3:**
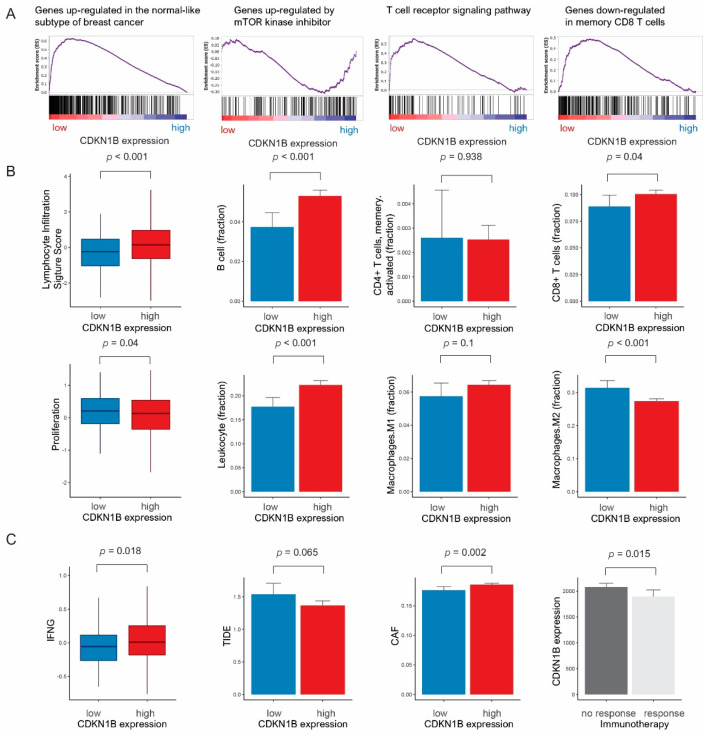
(**A**) In gene set enrichment analyses, CDKN1B expression is related to genes upregulated in the normal subtype of breast cancer, genes upregulated by mTOR kinase inhibitors, the T-cell receptor signaling pathway, and genes downregulated in memory CD8 T cells. (**B**) Bar plots: low CDKN1B expression is associated with a low lymphocyte infiltration signature score (*p* < 0.001), decreased B cells (*p* < 0.001), increased CD4+ T cells (*p* = 0.938), decreased CD8+ T cells (*p* = 0.04), high cell proliferation (*p* = 0.04), decreased leukocytes (*p* < 0.001), decreased M1 macrophages (*p* = 0.1), and increased M2 macrophages (*p* < 0.001). (**C**) Low CDKN1B expression is related to low interferon-g (IFNG) expression (*p* = 0.018), elevated Tumor Immune Dysfunction and Exclusion (TIDE) signature score (*p* = 0.065), and decreased cancer-associated fibroblasts (CAFs) (*p* = 0.002). The immunotherapy response was significantly different between low CDKN1B expression and high CDKN1B expression (*p* = 0.015).

In GSEA, low CDKN1B expression was associated with genes upregulated in the normal subtype of breast cancer, genes upregulated by mTOR kinase inhibitors, the T-cell receptor signaling pathway, and genes downregulated in memory CD8 T cells (Figure 3A).

Low CDKN1B expression was significantly correlated with low lymphocyte-infiltrating signature scores (*p* < 0.001), decreased B cells (*p* < 0.001), decreased CD8+ T cells (*p* = 0.04), high tumor cell proliferation (*p* = 0.04), low leukocyte count (*p* < 0.001), and elevated M2 macrophages (*p* < 0.001) (Figure 3B). Low CDKN1B expression was associated with low interferon-γ (*p* = 0.018), high TIDE signature score (*p* = 0.065), and decreased cancer-associated fibroblasts (*p* = 0.002). CDKN1B expression was decreased in patients with an anti-PD1 therapy response (*p* = 0.015) (Figure 3C). In our study cohort, a significant correlation was observed between low CDKN1B expression and reduced CD8+ T cell counts (*p* = 0.007). While low CDKN1B expression did not reach statistical significance, there was a correlation observed between low expression and a decline in the counts of CD4+ T cells (*p* = 0.254) (Supplementary Figure S1).

**Figure 4:**
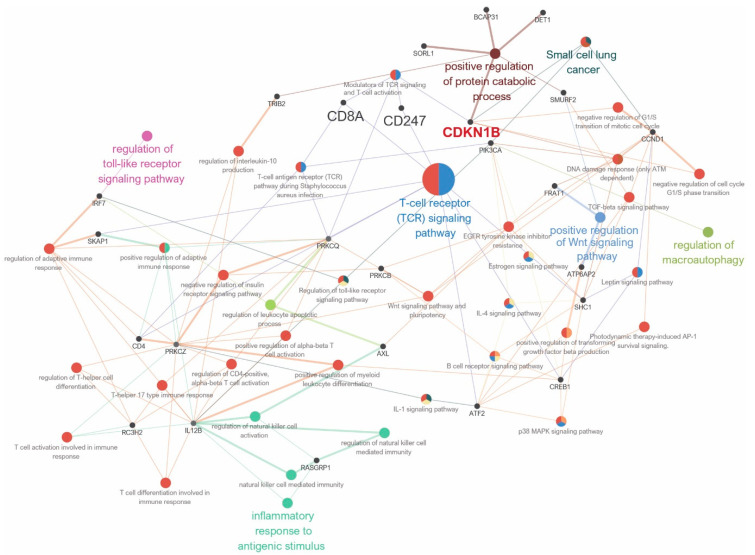
Grouping of networks according to functionally enriched Gene Ontology (GO) terms and pathways: CDKN1B (red) is directly or indirectly linked to the catabolic process, T-cell receptor signaling pathway, Wnt signaling pathway, and regulation of macroautophagy.

The pathway network analysis revealed direct links between CDKN1B and the protein catabolic process pathway. Furthermore, indirect connections were revealed between CDKN1B and pathways such as the T-cell receptor signaling pathway, the Wnt signaling pathway, the regulation of macroautophagy, the Toll-like receptor signaling pathway, and the inflammatory response to an antigenic stimulus (Figure 4).

### 3.3. Drug Screening and Machine Learning Analysis

**Figure 5:**
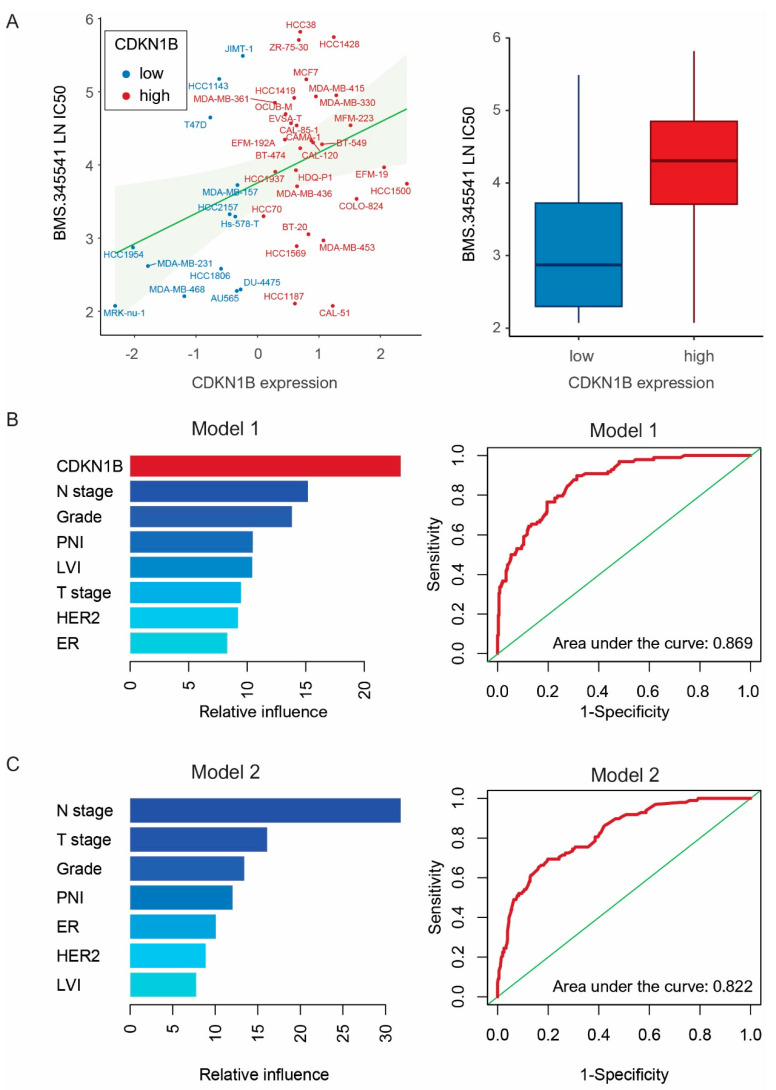
Genomics of Drug Sensitivity in Cancer (GDSC) database analysis. (**A**) Spearman’s correlations were performed to illustrate the relationships between the natural logarithm of the half-maximal inhibitory concentration (LN IC50) values obtained using BMS-345541 in breast cancer cell lines (r = 0.38, *p* = 0.013) (blue, low CDKN1B expression; red, high CDKN1B expression). Bar plot were performed to illustrate the LN IC50 values obtained using BMS-345541 between breast cancer cell lines with low (blue) and high (red) CDKN1B expression (*p* = 0.012) (error bars: standard errors of the mean). (**B**,**C**) A gradient boosting machine (GBM) model was employed to supervise a machine learning model for prognostic prediction within our 456 cohorts. Covariates were added in the confounding factors ((**B**) Model 1: T stage, N stage, histological grade (Grade), estrogen receptor (ER), human epidermal growth factor receptor 2 (HER2), perineural invasion (PNI), lymphovascular invasion (LVI), and CDKN1B versus (**C**) Model 2: T stage, N stage, Grade, ER, HER2, PNI, and LVI) and their relative importance using survival. A multivariate Bernoulli model was applied to generate a receiver operating characteristic curve for GBM.

BMS-345541 effectively inhibited the growth of breast cancer cells in 50 cell lines, especially those with low CDKN1B expression (*p* = 0.012). (Figure 5A). We assessed the predictive capability of two gradient boosting machine (GBM) models in determining survival rates in our cohort. The two GBM models were differentiated by the presence or absence of CDKN1B (Model 1 (CDKN1B, T stage, N stage, histological grade, lymphovascular invasion, perineural invasion, ER and HER2) versus Model 2 (T stage, N stage, histological grade, lymphovascular invasion, perineural invasion, ER and HER2)). The ROC curve for the GBM model was generated using the multivariate Bernoulli model. Adding CDKN1B to the prediction model improved the ability to predict prognosis, resulting in the GBM algorithm outperforming (area under the curve: Model 1, 0.869; Model 2, 0.822) (Figure 5B,C).

## 4. Discussion

This study investigated the potential effects of CDKN1B, a negative cell cycle regulator, in breast cancer. CDKN1B was found to primarily participate in anticancer functions, exhibiting lower expression levels in breast cancer tissue in comparison to normal breast tissue. Low CDKN1B expression has been shown to be significantly associated with advanced cancer stage and worse clinicopathological parameters, such as high expression of p53, Ki-67, and HER2. Furthermore, survival analyses in three databases (our cohort including HYGH and KBSMC, METABRIC, and TCGA) demonstrated that low CDKN1B expression is associated with unfavorable survival rates. Furthermore, low CDKN1B expression was related to various immune factors, such as low interferon-γ, CD8+ T cells, and immunotherapy response. According to our results, CDKN1B may represent a meaningful biomarker for breast cancer immunotherapy.

Cyclin-dependent kinase inhibitors (CDKIs) are critical negative regulators of cell cycle progression, and loss of cell cycle control may contribute to cancer development [32]. CDKN1B inhibits the enzymatic activity of the cyclin-CDK complex and plays a central role in the progression of the cell cycle from the G1 phase to the S phase. CDKN1B is highly expressed in all normal epithelia, including the breast, prostate, esophagus, stomach, colon, and lung mucous membranes. However, the loss of CDKN1B may occur in carcinomas containing these tissues [33]. Our results also revealed that CDKN1B expression was significantly lower in breast cancer tissues than in normal mammary gland tissues. Loss of CDKN1B function or decreased expression has been implicated in various cancer types, leading to uncontrolled cell cycle progression and increased tumor growth. Recent studies on the association of CDKN1B with breast cancer have identified CDKN1B as a driver gene that is almost exclusively mutated in luminal breast cancer (LBC) and found that it is enriched in mutations leading to loss of function in metastatic breast cancer patients [34]. Furthermore, high expression of CDKN1B is not only a marker of good prognosis but also serves as an independent predictor of response to hormonal therapy, while downregulation of CDKN1B has been shown to predict resistance to radiotherapy and anti-HER2 therapy [35,36]. Our study also found that reduced CDKN1B expression was linked to more aggressive tumor characteristics and poorer survival rates due to disease progression in breast cancer patients. This association was particularly strong in the luminal subtype. The precise molecular mechanisms and pathways responsible for carcinogenesis in breast cancer, as determined by CDKN1B expression, remain unclear.

The survival of cancer cells depends on the complex interactions between cancer cells and immune cells in the tumor microenvironment (TME). Marked lymphocyte infiltration by tumor-infiltrating lymphocytes (TILs) can play a central role in anticancer immunity in breast cancer patients and can be a beneficial prognostic factor in various cancers. Cytotoxic T lymphocytes (CTLs), which express CD8 on their cell surface, are prototypical antitumor immune cells and play an important role in anticancer immunity, as they can recognize tumor cells in an antigen-specific manner and directly kill them by secreting cytotoxic molecules [37]. An increase in the infiltration of CD8+ T cells is strongly associated with an improvement in OS in patients with ER-negative breast cancer. Better response to adjuvant chemotherapy is also associated with high immune infiltration [38,39,40]. Therefore, the identification of different types of immune cells in the tumor microenvironment provides an aid in predicting the prognosis of the cancer [41]. Using the GSEA, we observed a correlation between the expression of CDKN1B and specific gene sets associated with the T cell receptor signaling pathway and the downregulation of CD8+ T cells. These signaling pathways are important for the complete eradication of cancer cells. Our results showed that high CDKN1B mRNA levels were associated with higher lymphocyte-infiltrating signature scores and increased CD8+ T cells. These findings suggest that high CDKN1B expression promotes antitumor immune activity, leading to improved clinical outcomes. To support these findings, pathway network analysis using the TCGA database found that CDKN1B is indirectly involved in various immune pathways. These findings are in accordance with previous research [42,43].

CDKN1B may also have implications for therapeutic strategies in breast cancer. Targeting CDKs and cell cycle regulators has emerged as an attractive therapeutic approach in cancer treatment. By in vitro drug screening, we found that BMS-345541 effectively reduced breast cancer cells with low CDKN1B expression. BMS-345541, a potent small-molecule compound, is a highly selective inhibitor of IκB kinase (IKK)α and IKKβ, key regulators of NF-κB signaling [44]. Nuclear factor-κB (NF-κB) plays an important role in cell survival, proliferation, and differentiation and is often involved in malignant transformation [45]. NF-κB is activated primarily on T lymphocytes in response to T-cell receptor signaling but also in response to proinflammatory stimuli, where it protects tumor cells from death in many cancers, thereby influencing tumor development and cancer treatment resistance [46,47]. Further studies are needed to evaluate the efficacy of these treatments in specific subsets of breast cancer patients based on CDKN1B expression.

In this study, we used GBM analysis to investigate the influence of CDKN1B expression on breast cancer survival. GBM, a machine learning technique, has the advantage of processing many predictor variables through a simple prediction algorithm. It combines the results in a non-linear and interactive way, thereby increasing the accuracy of the predictions. The CDKN1B-induced survival model improvement suggests that CDKN1B is a potent prognostic factor in breast cancer.

The present study has several limitations. First, the detailed molecular mechanisms and signaling pathways through which CDKN1B influences the clinical outcomes of breast cancer, using our bioinformatics approach and in silico analysis, need to be experimentally validated. Specifically, the proposition that CDKN1B could serve as a biological marker for anticancer immunotherapy, as assessed by the TIDE tool, requires validation across multiple research cohorts. Second, the breast cancer data from TCGA and METABRIC did not include sufficient clinicopathologic parameters to build a machine-learning model to predict survival. This study could not assess the impact of CDKN1B due to the limited data available. Third, the statistical significance of the difference in CD8+ T cell counts between cases with low and high CDKN1B expression was observed in a limited number of cases using immunohistochemistry. Fourth, we did not consider ki67 in classifying breast cancer subtypes in this study. Further studies with larger cohorts will be essential to validate our findings. Fourth, the drugs proposed in this study may be different depending on the immune response and disease status. Therefore, our results need to be further verified.

## 5. Conclusions

Our findings provide insights into the potential role of CDKN1B as a biomarker for survival prediction and immunotherapy response in breast cancer. Low CDKN1B expression is associated with low lymphocyte-infiltrating signature scores and decreased CD8+ T cells, which could induce unfavorable physiological changes. Intriguingly, there is a correlation between low CDKN1B expression and immunotherapy responsiveness. In vitro drug screening showed that BMS-345541 reduces the growth of breast cancer cells with low CDKN1B expression. CDKN1B may serve as an important biomarker in the treatment of breast cancer, and further experimental research and clinical trials of targeted drugs for CDKN1B may be necessary to clarify its clinical significance. Our results are expected to play an important role.

## Figures and Tables

**Table 1:** Clinicopathological parameters of p27 (encoded by CDKN1B gene) expression in our cohort.

Parameter	CDKN1B Expression		*p*-Value ^1^
	Low (*n* = 85), *n* (%)	High (*n* = 371), *n* (%)	
Age (years)	50.4 ± 10.0	49.3 ± 9.9	0.333
T stage			
1	30 (35.3%)	178 (48.0%)	**0.034 ^3^**
2	48 (56.5%)	178 (48.0%)	
3	7 (8.2%)	15 (4.0%)	
N stage			
0	39 (45.9%)	194 (52.3%)	**0.021 ^3^**
1	22 (25.9%)	113 (30.5%)	
2	15 (17.6%)	45 (12.1%)	
3	9 (10.6%)	19 (5.1%)	
Histological grade			
1	13 (15.3%)	74 (19.9%)	0.325 ^3^
2	45 (52.9%)	171 (46.1%)	
3	27 (31.8%)	126 (34.0%)	
Lymphatic invasion			
Negative	44 (51.8%)	186 (50.1%)	0.88
Positive	41 (48.2%)	185 (49.9%)	
Vascular invasion			**0.001**
Negative	72 (84.7%)	354 (95.4%)	
Positive	13 (15.3%)	17 (4.6%)	
Perineural invasion			**0.008**
Negative	58 (68.2%)	304 (81.9%)	
Positive	27 (31.8%)	67 (18.1%)	
ER			0.356
Negative	28 (32.9%)	101 (27.2%)	
Positive	57 (67.1%)	270 (72.8%)	
PR			0.44
Negative	37 (43.5%)	142 (38.3%)	
Positive	48 (56.5%)	229 (61.7%)	
HER2			**0.016**
Negative	69 (81.2%)	249 (67.1%)	
Positive	16 (18.8%)	122 (32.9%)	

T or N stage, 8th edition; ER, estrogen receptor; PR, progesterone receptor; HER2, human epidermal growth factor receptor 2. ^1^ Chi-square test. ^3^ T stage: 1 vs. 2, 3; N stage: 0, 1 vs. 2, 3; Histological grade: 1 vs. 2, 3. *p* < 0.05 is shown in bold.

**Table 2:** Overall and disease-free survival analyses according to p27 (encoded by CDKN1B gene) from our cohort.

Covariate	Disease-Free Survival				Overall Survival			
	HR	95%CI		*p*-Value	HR	95%CI		*p*-Value
Our cohort								
Multivariate ^1^	0.530	0.330	0.852	0.009	0.498	0.315	0.788	0.003
Covariate	Disease-free survival				Disease-specific survival			
	HR	95%CI		*p* value	HR	95%CI		*p* value
TCGA								
Multivariate ^2^	0.560	0.318	0.989	0.046	0.509	0.267	0.971	0.04
Covariate	Disease-specific survival				Overall survival			
	HR	95%CI		*p* value	HR	95%CI		*p* value
METABRIC								
Multivariate ^3^	0.829	0.689	0.999	0.048	0.857	0.746	0.984	0.028

^1^ Adjusted for T stage, N stage, estrogen receptor, histological grade, lymphatic invasion. ^2^ Adjusted for T stage, N stage, estrogen receptor, histological grade. ^3^ Adjusted for T stage, N stage, estrogen receptor, histological grade.

## Data Availability

Public data used in this work can be acquired from the portal TCGA Research Network (https://gdc.cancer.gov/about-data/publications/pancanatlas) (accessed on 1 June 2021). The data presented in this study can be available on request from the corresponding author. The data are not publicly available due to privacy.

## Supplementary Materials

The following supporting information can be downloaded at: https://www.mdpi.com/article/10.3390/jpm14010030/s1, Figure S1: Correlation of CD8+ T cell count and CD4+ T cell count with CDKN1B expression; Table S1: Disease-free and disease-specific survival analyses of p27 (encoded by CDKN1B gene) based on subtype.
