# Supplementary material for: Low CDKN1B Expression Associated with Reduced CD8+ T Lymphocytes Predicts Poor Outcome in Breast Cancer in a Machine Learning Analysis
Source: J Pers Med. 2023 Dec 25;14(1):30. doi: 10.3390/jpm14010030 (PMC10817603; doi:10.3390/jpm14010030)
Supplement: Supplementary file 1 [file jpm-14-00030-s001.zip › supplementary table1.pdf]

Supplementary table 1. Disease-free and disease-specific survival analyses of p27 (encoded by CDKN1B gene) based on subtype.

Covariate	Disease-free survival				Overall survival			
	p value	HR	95%CI		*p* value	HR	95%CI	
HR(+)HER2(-)								
Univariate	**0.014**	0.504	0.292	0.869	**0.003**	0.449	0.263	0.766
Multivariate[1]	**0.043**	0.563	0.323	0.981	**0.033**	0.556	0.324	0.954
HR(+)HER2(+)								
Univariate	**0.012**	0.237	0.078	0.725	**0.032**	0.336	0.124	0.913
Multivariate[1]	0.131	0.365	0.099	1.349	0.061	0.346	0.114	1.049
HR(-)HER2(+)								
Univariate	0.371	0.567	0.163	1.969	0.109	0.398	0.129	1.230
Multivariate[1]	0.489	0.623	0.163	2.381	0.196	0.424	0.115	1.557
HR(-)HER2(-)								
Univariate	0.123	0.307	0.068	1.379	0.316	0.489	0.121	1.977
Multivariate[1]	0.276	0.411	0.083	2.034	0.402	0.541	0.129	2.278

HR, hormone receptor; HER2, human epidermal growth factor receptor 2

[1] Adjusted for T stage, N stage, histological grade, lymphatic invasion
