# Supplementary figures and images for: Low CDKN1B Expression Associated with Reduced CD8+ T Lymphocytes Predicts Poor Outcome in Breast Cancer in a Machine Learning Analysis
Source: J Pers Med. 2023 Dec 25;14(1):30. doi: 10.3390/jpm14010030 (PMC10817603; doi:10.3390/jpm14010030)

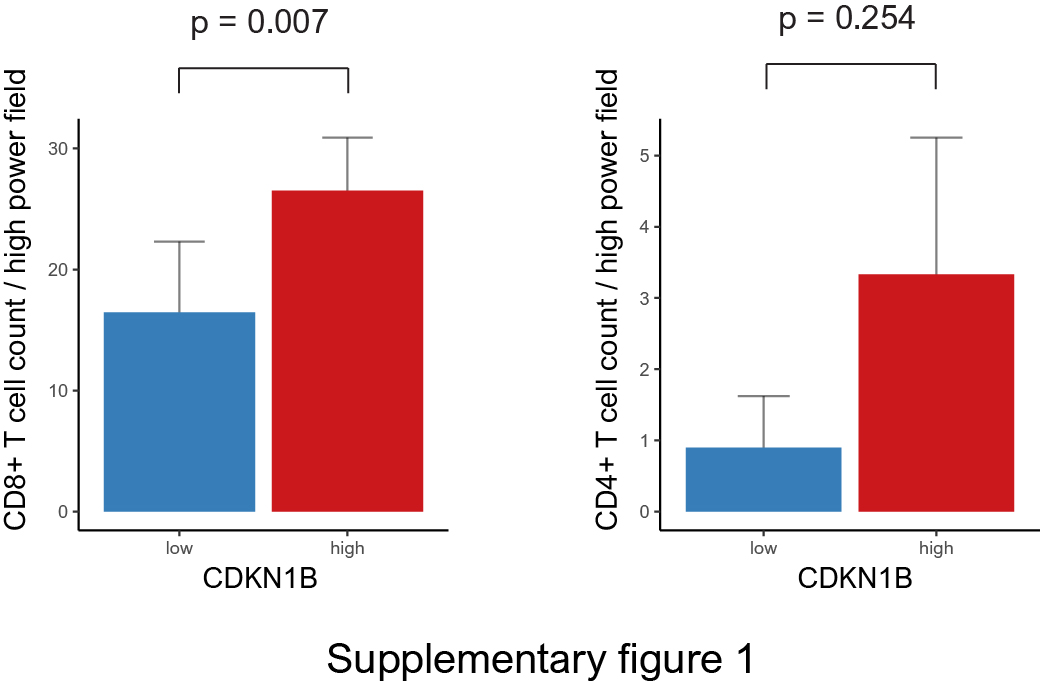

Supplement: Supplementary file 1 [file jpm-14-00030-s001.zip › Supplementary figure 1.jpg]
